# Prof. Samuel Jackson, M.D., of Philadelphia

**Published:** 1872-04-01

**Authors:** 


### Obituary.

### PROF. SAMUEL JACKSON, M.D.,

OF PHILADELPHIA.

Dr. Samuel Jackson, Emeritus Professor of the Institute of Medicine in the University of Pennsylvania, died, April 6, at his residence, in this city, at the ripe old age of eighty-two. He was born in Philadelphia on the 22nd of March, 1787. He devoted his time with great energy to the study of his profession, and was successful in acquiring a high standing in its practice. In 1835 he was elected Professor of the Institute of Medicine in the University of Pennsylvania, a position which was filled by him during a period of twenty-eight years, with great credit both to himself and the institution. He was one of the clearest lecturers and profoundest scholars and occupied a front rank in the corps of Professors of the University, a body of men which has long enjoyed the highest reputation for skill and learning throughout the world. In 1863, in consequence of his advanced age, he abandoned the active duties of his Professorship, and has since held an Emeritus relation to the University. Prof. Jackson wrote several works on medical and physiological subjects, the most celebrated of which were a treatise on the "Principles of Medicine," first published in 1832, and an "Introduction to Lehman's Chemical Physiology," published in 1856.—*Philadelphia Inquirer.*

Delay in appearance of the *Journal* enables us to announce the death, upon April 5th, of Zina Pitcher, M.D., of Detroit, Mich. Dr. Pitcher was aged about 75 years, was one of the oldest citizens, assisted in founding the free schools of Detroit, and the State University, of which he was for many years a regent. He had occupied many prominent secular and professional positions. He was an Emeritus Professor in the Michigan University, a position to which he was nominated by the present writer. He had also been one of the Presidents of the American Medical Association. He was a model of the courtly gentleman of the old school, and his death will be widely lamented.—*Chicago Medical Journal.*

Prof. Samuel Henry Dickson, M.D., LL.D., died in Philadelphia, March 31, 1872.

